# Physicochemical, Morphological, Thermal, and Rheological Properties of Native Starches Isolated from Four Cultivars of Anchote (*Coccinia abyssinica* (*Lam.*) *Cogn.*) Tuber

**DOI:** 10.3390/gels8090591

**Published:** 2022-09-16

**Authors:** Yohannes Tolesa Wolde, Shimelis Admassu Emire, Workineh Abebe, Felicidad Ronda

**Affiliations:** 1 School of Chemical and Bio-Engineering, Addis Ababa Institute of Technology, Addis Ababa University, King George VI Street, Addis Ababa P.O. Box 385, Ethiopia; 2 Department of Agriculture and Forestry Engineering, Food Technology, College of Agricultural and Forestry Engineering, University of Valladolid, 34071 Valladolid, Spain; 3 Ethiopian Institute of Agricultural Research, Addis Ababa P.O. Box 2003, Ethiopia

**Keywords:** anchote tuber, starch, gels, physicochemical properties, pasting properties, thermal properties, rheological properties

## Abstract

Anchote (*Coccinia abyssinica* (*Lam*.) *Cogn*) is a potentially important source of starch and an underutilized root and tuber crop indigenous to Ethiopia. In this study, the physicochemical, morphological, thermal, and rheological properties of native starches isolated from four cultivars of anchote tubers were studied and compared to potato and cassava starches, which were considered as references. The amylose content of anchote starches varied from 15.8–22.3%. The anchote cultivars showed different granule sizes, but all revealed a B-type crystalline structure, identical to potato starch. The phosphorus content of anchote starches ranged from 82–93 mg/100 g and was much higher than that of potato and cassava (60.3 and 5.8 mg/100 g, respectively). This characteristic could govern several functional properties of anchote starches, making them suitable for applications in different types of noodles, glucose syrups, and viscous products. The gelatinization temperature and enthalpy of anchote starches, which ranged from 60.97 °C to 69.33 °C and 16.87 to 18.38 J/g, respectively, were considerably different compared to potato and cassava starches. Significant variations were also observed among the pasting properties of starches from anchote cultivars. They showed a higher stability to heating and shearing, having higher TV (2046 to 2280 mPa·s) and lower BV (248 to 487 mPa·s) values, and a higher final viscosity (3409 to 3686 mPa·s) than potato and cassava, which are important characteristics in food processing and when high gel viscosity is required after cooling. Anchote starch gels exhibited rheological characteristics of true gels, showing much lower (tan δ)_1_ values and significantly higher viscoelastic moduli than those found in cassava and potato gels. The present study revealed significant differences among the physicochemical properties of anchote starches, depending on the cultivar, and demonstrated their promising potential in food product development and other industrial applications.

## 1. Introduction

Starch constitutes a significant component of our diet and has a variety of industrial applications [1]. Despite the increasing annual world consumption, the main sources of starch have been limited to a few crops, such as corn, potato, wheat, cassava, and sweet potato. Hence, researchers have been prompted to investigate the physicochemical and functional characteristics of starches extracted from various genotypes and botanical sources. Recently, researchers have shown an increased interest in the extraction of starches from underutilized root and tuber crops, as they contain an abundant amount of carbohydrates mainly starch [1,2,3].

Anchote (*Coccinia abyssinica* (*Lam.*) *Cogn*) is an indigenous tuber crop grown in Ethiopia for economic, nutritional, medical, and socio-cultural benefits [1,4]. It is an annual trailing vine, categorized under root and tuber crops, that belongs to the order *Cucurbitales* and the family *Cucurbitaceae.* Anchote cultivation and utilization have been a common practice in western parts of Ethiopia, mostly in the Wollega, Illu-Abba Bora, and Jimma areas, but recently, its use has expanded to the southern and southwestern areas of the country [5,6,7], and the tuber is relatively new to the rest of the globe, with minimal scientific information [4]. It has been reported that the anchote tuber contains a substantial amount of valuable nutrients, including carbohydrates, protein, fiber, fat, and various minerals, with a total solids content that ranges from 25–28.5 g/100 g [8,9]. The tuber is known for its high protein and calcium content compared to other root and tuber crops [6,8]. Anchote tuber is rich in starch content (78.71–75.56%) [1,5,7], making it a viable alternative source of starch for application in a variety of fields. However, understanding the physicochemical properties of starches obtained from such underutilized roots and tubers is important for the development of value-added novel products. The use of starch in the food and non-food sectors is determined by its physicochemical quality, which includes morphology, particle size, amylose contents, crystallinity, thermal properties, swelling powers, and hydrolysis capabilities [2,10]. These properties are key factors, as they dictate their functionality in different applications. The physicochemical properties of starches obtained from different crops have been reported. For example, a novel annatto seed starch showed promising characteristics compared to commercial corn starch and it can be used in heat sensible foods [11]; physicochemical properties of ramie starch reveal its potential utilization as a thickening agent, and it can be used alternatively to potato starch in food and non-food industries [3]; lycoris starches exhibited higher amylose content than common cereal starches, which is its unique characteristic [12]; the variability in starch granule size, amylose, protein, and lipid contents observed in cassava varieties greatly influences the physicochemical properties of starches [13], and the variety and relationships obtained between the physicochemical properties of different sweet potato starches predict their potential applications in food and non-food products [2,14].

Anchote tubers include both red and white tissue colors [8]. Bikila et al. [4] evaluated the effect of pre-drying treatment and drying temperature on proximate composition, mineral contents, and thermophysical properties of anchote flour obtained from the first released anchote variety (Desta 1) by the Ethiopian Institute of Agriculture Research. However, to date, there is no scientific documentation on anchote starch properties from different genotypes, or the rheological properties of anchote gels, which are so important for their food applications. Thus, in this study, the native starches isolated from four cultivars of anchote tuber were characterized. The study covered the physicochemical, morphological, structural, pasting, rheological, and thermal properties of native starches isolated from four anchote cultivars (one variety and three cultivars) grown in Ethiopia. Commercial potato and cassava starches were included as references for comparison. The findings of this study will provide scientific information for further utilization of anchote starches obtained from diverse cultivars as functional ingredients, as well as their intended applications in food product development and other industrial applications.

## 2. Results and Discussion

### 2.1. Starch Particle Size and Morphology

The particle size of starch samples was measured as surface- and volume-weighted mean diameter. The mean diameter (D_50_) of all starch samples varied in the following order: D01S (11.58 μm) < REDS (13.31 μm) < CS (13.45 μm) < D24S (14.41 μm) < WHTS (14.48 μm) < PS (36.21 μm) (Table 1). The D_50_ values of all anchote starches were smaller than that of PS, while they were comparable to that of CS. The size dispersion (D_90_ − D_10_)/D_50_) of anchote starch (15.45–22.47) were considerably higher than those of PS (1.27) and CS (1.0) starches. The surface- (D [3,2]) and volume-weighted mean diameter (D [4,3]) of anchote starches varied from 12 μm (D01S) to 14.7 μm (WHTS) and 63.9 μm (D01S) to 106.9 μm (WHTS), respectively. Significant differences (*p* < 0.05) in all parameters were observed among the four cultivars of anchote, potato, and cassava starches. The differences in the particle size of the starches are likely attributed to cultivar differences, growing conditions, and plant physiology [2]. According to the classification of Lindeboom, Chang, and Tyler [15], the particle size of anchote starch fits into the medium-sized group. Starch particle size and size distribution play a significant role in influencing the functionality and intended applications of starches.

**Figure 1:**
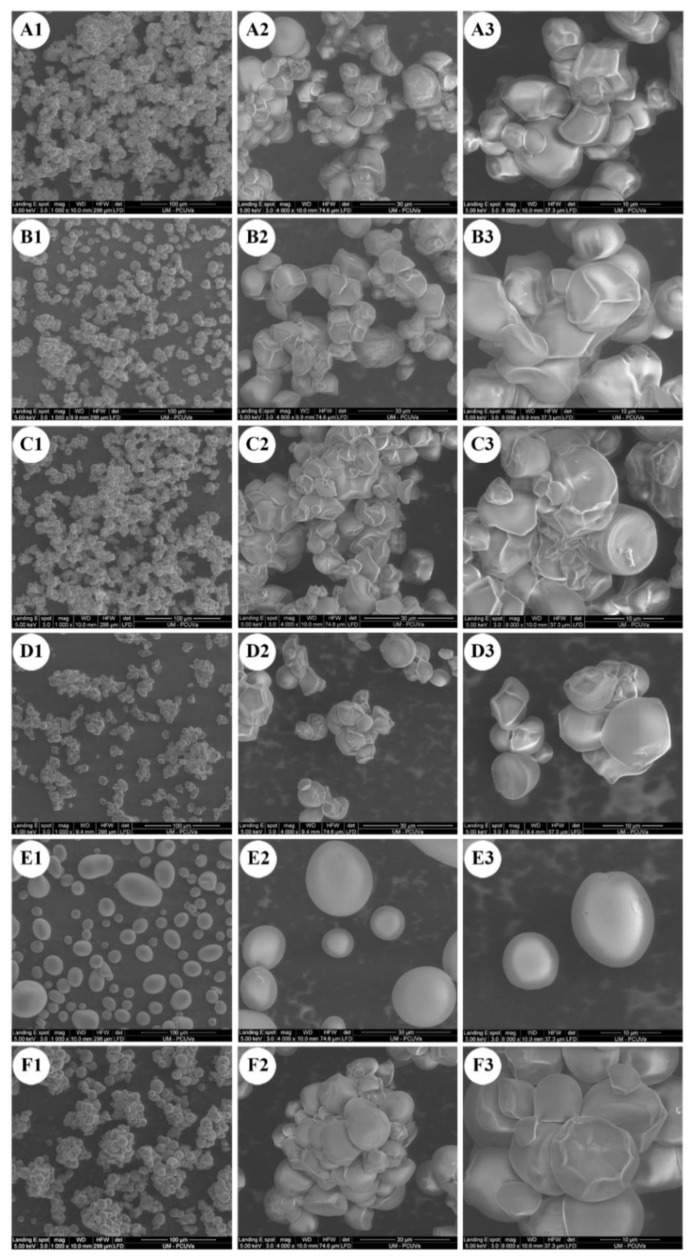
SEM images of starches from four cultivars of anchote (**A**–**D**), potato starch (**E**), and cassava starch (**F**); (**A**) D01S = Desta 01 starch; (**B**) D24S = Desta 24 starch; (**C**) WHTS = white starch; (**D**) REDS = red starch; (**E**) PS = potato starch; (**F**) CS = cassava starch, at magnifications of (1) 1000×, (2) 4000×, and (3) 8000×.

The four cultivars of anchote, potato, and cassava starches showed significantly different morphologies, as observed from scanning electron micrographs (Figure 1). Anchote starch granules showed a mixture of various shapes and sizes. The anchote starch granules were polygonal, dome-shaped, and irregular in shape, containing a mixture of small and large granules clustered together and having a smooth surface. The studied anchote cultivars had similar granule morphologies, but different extents of clustered granules. On the other hand, PS and CS starches revealed diverse populations of large, medium, and small granules, showing ellipsoidal and spherical shapes for potato, and oval, oval-truncated, round, polygonal, and round polygonal for cassava starch, which was in agreement with previous reports [16,17,18,19]. The differences in granule morphology of anchote, potato, and cassava starches could be due to the differences in the physiology of the plant, as well as the biological origin and biochemistry of the amyloplast [20].

**Figure 2:**
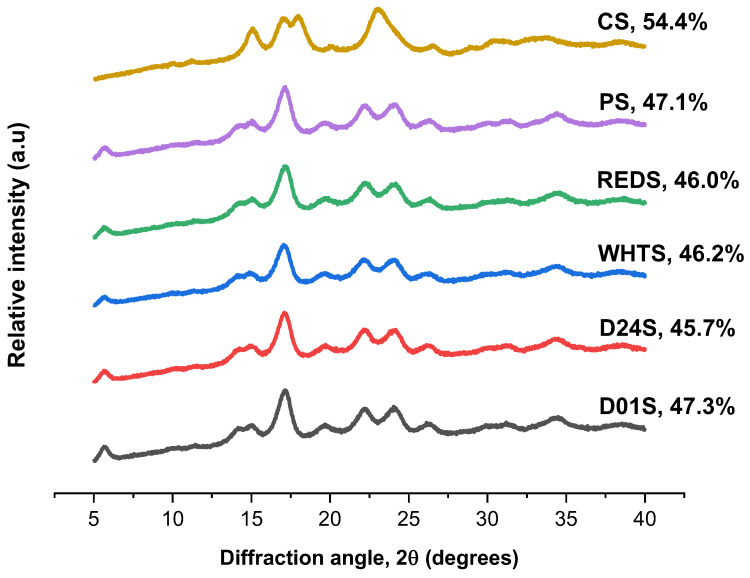
The XRD pattern of starches from four cultivars of anchote. Potato and cassava starches are included as references. D01S = Desta 01 starch, D24S = Desta 24 starch, WHTS = white starch, REDS = red starch, PS = potato starch, and CS = cassava starch. The degree of crystallinity for all samples is shown on the graph.

The X-ray diffraction pattern and relative crystallinity of the studied starches are shown in Figure 2. All anchote cultivars showed similar diffraction peaks, with the greatest intensity at around 5.6°, 15°, 17°, 22°, and 24° of 2θ, which is characteristic of the B-type diffraction pattern. This result was consistent with previous studies reported by Abera et al. [5]. However, there were slight differences in the intensities of diffraction peaks between the four cultivars of anchote starches. PS also presented a B-type diffraction pattern, while CS showed strong diffraction peaks at around 15° and 23° of 2θ, and a doublet at 17° and 18° of 2θ, which is characteristic of the A-type diffraction pattern. The results obtained agreed with previous works [3,5,18]. The relative crystallinity of the four starches obtained from anchote cultivars was in the range of 45.7 (D24S) and 47.3% (D01S) (Figure 2). Comparatively, CS starch (54.4%) had higher relative crystallinity than anchote starches, whereas PS showed a nearly similar value with anchote starches. The relative crystallinity of anchote starches obtained in this study was higher than the previous report [7], which could be due to the difference in moisture content of the studied samples; it was reported that the crystallinity was directly proportional to the amount of water present in the sample, i.e., the crystallinity increased with an increase in hydration [13,21]. The crystalline structure of starch is affected by several factors, such as growing environment, crystal size, packing density inside the granules, length of the amylopectin chain, orientation of the double helices inside the crystalline area, and moisture content [22,23]. Moreover, the degree of crystallinity is inversely proportional to the amylose content [10], which was in agreement with the findings of this study.

### 2.2. Proximate Composition

The proximate compositions of anchote, potato, and cassava starches are shown in Table 2. The moisture contents of starches isolated from anchote cultivars varied significantly (*p* < 0.05), and D01S has higher moisture content compared to other anchote cultivars. The ash contents of anchote starches varied between 0.38% and 0.49% and were significantly higher than those recorded in PS and CS starches. The D24S had the highest fat content (0.14%), while the rest of the anchote starches had comparable fat content to PS (0.09–0.11%), and CS showed significantly lower fat content compared to all studied starch samples.

The protein content of the anchote starches varied between 0.51% (WHTS) and 0.65% (D01S) (Table 2) and was significantly higher (*p* < 0.05) than that of either the PS or CS starches. The protein content in tuber starch relies on the species and variety of starch [14]. The higher protein content obtained in anchote starches might be due to the high protein content of the anchote tuber, which makes it unique when compared to other root and tuber crops [4]. The results obtained in this study were higher than the findings reported by Abera et al. [5]. The high protein content obtained in this study could be due to the varying botanical origin, different starch isolation techniques, and differences in their genotype backgrounds.

### 2.3. Mineral Content

Phosphorus is an important factor in several functional properties of starch, which in turn govern its aptness for the desired application [24]. The phosphorus content of the studied starches from anchote cultivars ranged between 82.8 mg/100 g (D01S) and 93.3 mg/100 g (WHTS) (Table 2), which was significantly higher than that of the reference starches, e.g., PS (60.3 mg/100 g) and CS (5.8 mg/100 g). To date, there is no published literature on the phosphorus content of anchote starch; however, the phosphorus content of PS and CS starches were reported by various scholars, in agreement with the result in this study [16,25]. Noda et al. [25] reported that the phosphorus content of different cultivars of potato starch varied between 57.9 and 98.1 mg/100 g. Low values of phosphorous content were reported for cassava starch [16]. The phosphorus content could be influenced by growing conditions, temperature, and storage [26]. The high phosphorous content in anchote starch could make it suitable for applications in different types of noodles, glucose syrups, and viscous products, as well as in the formulation of various foods, as it favors the functional characteristics of the starch by enhancing paste viscosity, clarity, and lightness [24]. The calcium content of anchote starches isolated from four cultivars varied significantly (*p* < 0.05), between 30.4 mg/100 g (WHTS) to 55.7 mg/100 g (D24S) (Table 1). The PS and CS starches exhibited significantly lower calcium contents compared to anchote starches. The higher calcium content observed in anchote starch could be due to the presence of an ample amount of calcium in the anchote tuber [4].

### 2.4. Amylose and Total Starch Contents

The total starch content of anchote samples varied from 66.8% (REDS) to 80.8% (D01S) (Table 2) and was relatively lower than that found in PS (84.1%) and CS (92.2%). The lower starch content obtained in anchote starch samples, and in particular in the REDS cultivar, denotes the higher amount of other non-starch carbohydrates (possibly fiber) retained with starch during the isolation process. Abera et al. [5] reported a 78.7% starch content of anchote starch isolated from unidentified cultivars, which was in the range of our results. The amylose content of the starches isolated from the four anchote cultivars showed significant (*p* < 0.05) variations, ranging from 15.8% (D01S) to 22.3% (D24S) (Table 2). High amylose starches are suitable for making pasta, sweets, bread, and as a coating material in fried products due to their high gelling strength [2]. The amylose contents obtained in anchote starch were within the range reported for most tuber and root starches and therefore, it can be categorized as a regular starch [16,21]. Likewise, the amylose contents of PS and CS were 18.8% and 19.0%, respectively, which was lower than the amylose contents of D24S and REDS, while it was higher than the D01S and WHTS anchote cultivars. The amylose contents of anchote starches observed in this study were lower than the amylose contents reported by Tessema et al. [7], which could be due to the fact that amylose content is greatly affected by the genotype background, growing environment, and measuring method [14]. Tessema et al. [7] used a starch-iodine colorimetric method (with known positive interference of the long amylopectin changes), while we used a method that includes a prior separation step using concanavalin-A (Con-A), which specifically complexes branched polysaccharides of amylopectin starch components [27].

### 2.5. Pasting Properties

**Figure 3:**
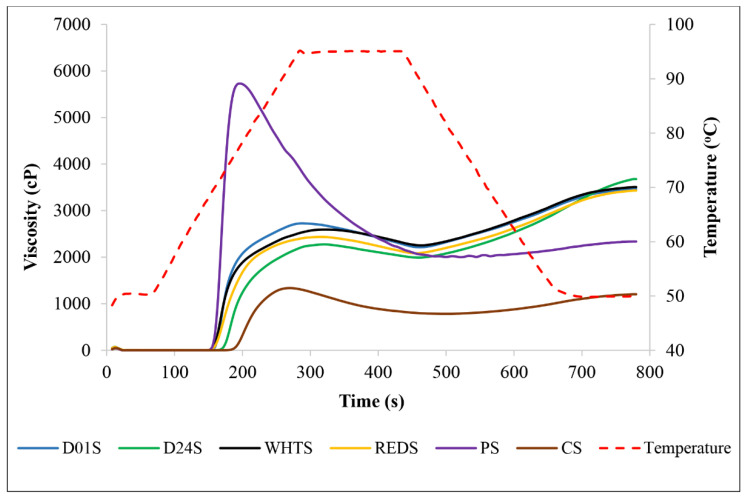
Viscometric profiles of starches from four cultivars of anchote (D01S = Desta 01 starch, D24S = Desta 24 starch, WHTS = white starch, REDS = red starch). Potato (PS) and cassava (CS) starches are included as references.

The pasting properties play a crucial role in defining the quality and utilization of starch for various applications in the industry [2,12,14]. The pasting parameters patterns of anchote, potato, and cassava starches are presented in Table 3 and Figure 3. The PV of the anchote cultivars were varied as follows: 2293 mPa·s (D24S) < 2448 mPa·s (REDS) < 2649 mPa·s (WHTS) < 2726 mPa·s (D01S). The PV values of the four anchote cultivar starches were two times higher than that of the CS and considerably lower than that of PS. The highest values of TV were obtained in WHTS (2280 mPa·s) and D01S (2239 mPa·s) and the lowest in D24S (2046 mPa·s) and REDS (2058 mPa·s). In all cases, regardless of cultivar, the TV of anchote starch was much higher than that of CS (796 mPa·s). TV is an important factor for starch processing because it distinguishes the ability of starch to withstand heating and shear stress [12]. According to Aina et al. [28], starches with high TV would be ideal in applications that require high starch consistency during prolonged cooking. Thus, from the findings of this study, the anchote starch could be the best candidate for industrial applications which require higher cooking time. BV reveals the property of the starch paste to resist heating and shearing. A high BV value means low stability of the paste viscosity under heating and stirring [2]. The four cultivars of anchote starch had lower BV values than the PS and CS starches; thus, anchote starch showed the best resistance and stability. The lowest BV value was obtained from the D24S (248 mPa·s), while the highest corresponded to D01S (487 mPa·s), which was not significantly different from that of CS. It can be concluded that anchote starch had the best resistance to heating and shearing, and in particular, the D24S would be the most stable. The SB of anchote starch, which ranged from 1215 mPa·s (WHTS) to 1640 mPa·s (D24S), was significantly higher (*p* < 0.05) than that of PS (380 mPa·s) and CS (426 mPa·s). SB is related to the retrogradation tendency, or gelling ability, of amylose [29]. Thus, the higher setback viscosities obtained in this study suggest a higher retrogradation tendency of anchote starches when compared to PS and CS. FV, the paste viscosity obtained at the end of cooling at 50 °C, indicates the capacity of starch to form a paste or gel after cooling, indicating the stability of the cooked paste [30]. In this study, the FV of anchote starch varied between 3409 mPa·s (REDS) and 3686 mPa·s (D24S), which was significantly higher (*p* < 0.05) than that of the PS (2322 mPa·s) and CS (1222 mPa·s) starches. Thus, the FV of anchote starch suggested the higher stability of the gel after cooling and the suitability for applications which require higher final viscosities. The PT of the starches from the anchote cultivars varied significantly (*p* < 0.05) among themselves, with the highest value obtained from the D24S (72.65 °C), followed by D01S and WHTS, while REDS scored the lowest value (69.50 °C). The PT of the anchote starches fell between those of the reference starches, PS (68.8 °C) and CS (75.65 °C). The highest Ptime was obtained in the REDS (5.15 min), while the lowest was recorded in D01S (4.89 min). In all anchote starches, the Ptime was higher than in PS (3.27 min) and CS (4.47 min). The high content of phosphate groups in the anchote starches can influence their pasting properties and contribute to the high peak viscosities obtained, regardless of the cultivar [24]. However, many other characteristics affect the pasting properties of anchote starch. Generally, it is difficult to associate the pasting characteristics of the starches across studies because of fact that the variances in chemical constituents and amylose content, the structure of amylose and amylopectin, granule size, crystal structure, and differences in botanical sources greatly impact the pasting properties of starch [2,31].

### 2.6. Rheological Properties

**Figure 4:**
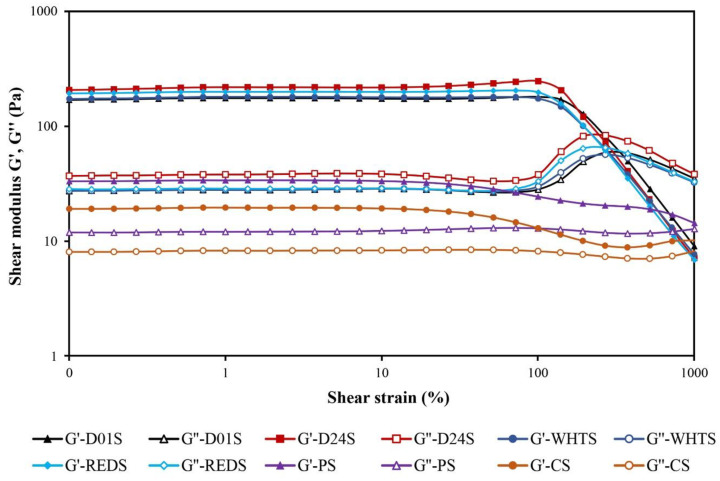
Strain sweeps of gels made with starches from four cultivars of anchote (D01S = Desta 01 starch, D24S = Desta 24 starch, WHTS = white starch, REDS = red starch), potato starch (PS), and cassava starch (CS). The elastic modulus, G′, is represented using solid symbols and the viscous modulus, G″, with open symbols.

The rheological properties of the starch gels formed from four cultivars of anchote were investigated and compared to the starch gels made from PS and CS starches (Table 4). The parameters obtained by fitting the power-law model to the frequency sweep data (from 1 to 10 Hz) of the studied starch gels are shown in Table 4. The maximum stress the samples can tolerate in LVR (τ_max_) and the stress at the cross point (G′ = G′′ and tan δ = 1) obtained from the strain sweep tests were also recorded. The strain sweeps were carried out to establish the linear viscoelastic region (LVR), and two different regions were observed in the four cultivars of anchote starch gels (Figure 4): the LVR region, in which G′, G′′, and tan δ values were almost constant, and the non-linear area, in which G′ started to decrease faster than G′′, and consequently, the (tan δ)_1_ began to increase with increasing strain until the curves of G′ and G′′ intersected (G′ = G′′ and tan δ = 1). The cross point is a singular and interesting point that can be considered as a yield stress. It represents the stress where the rheological behavior of the gels changes from a viscoelastic solid to that of an elasto-viscous liquid [32]. It is a value well correlated with the gel structure stability although sometimes, in very structured gels, this value is not achieved, as occurs in the case of PS and CS gels. For PS and CS gels, the tan δ was below 1 over the entire studied strain range (0.10 to 1000%), which reveals the high stability of these gels. The same result was observed for their τ_max_ values within the LVR: it was not possible to establish them for the PS and CS gels due to the small drops observed in the G’ and G’’ moduli during their strain sweeps over the entire studied range. This finding was in agreement with the finding in previous studies [33].

The starches from the four anchote cultivars showed significant variations in their maximum stress (τ_max_) resisted by the gels before the disruption of their structure, which occurred at the end of the LVR. The highest τ_max_ (181 Pa) was obtained in D01S anchote starch, while the lowest τ_max_ (131 Pa) was obtained in WHTS anchote starch. The cross point (G′ = G′′ and tan δ = 1) of anchote starch varied from 242 Pa (REDS) to 285 Pa (D01S). Therefore, the stability of the gels made with starches from the four anchote cultivars was lower than that of the PS and CS gels. According to the values of τ_max_ and the stress at the cross point (Table 4), it can be concluded that the most stable anchote gels were those made from D01S and D24S, while the least stable were those made from WHTS and REDS.

**Figure 5:**
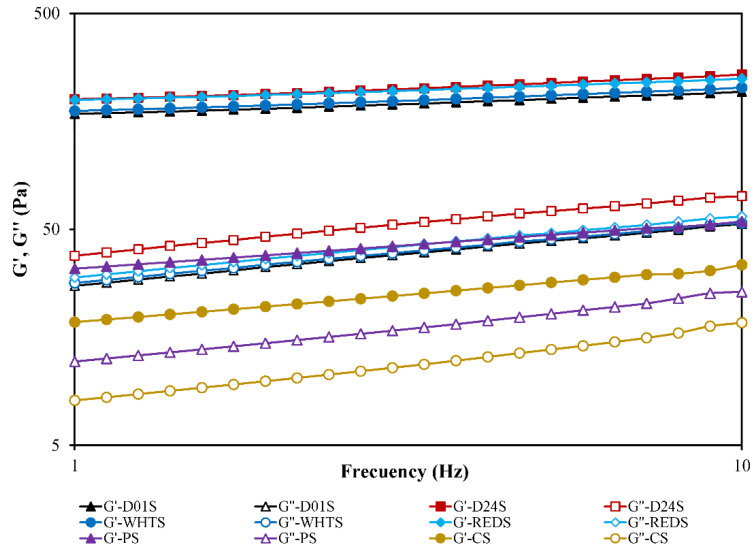
The mechanical spectra curve of anchote starch gels (D01S = Desta 01 starch, D24S = Desta 24 starch, WHTS = white starch, REDS = red starch), potato starch (PS), and cassava starch (CS) obtained from frequency sweep tests (G′ and G″ versus frequency). G′ is represented using solid symbols, and G″ with open symbols.

The viscoelastic behavior of starch was studied by measuring the evolution with the frequency of G′ (storage modulus) and G″ (loss modulus), which reveal elasticity and viscosity respectively, and tan δ (loss tangent), which quantifies the ratio between them (G’’/G’). Frequency sweeps (Figure 5) showed that G′ increased over the frequency range and the values of G′ were higher than that of G″ for all the starch gels studied, with tan δ < 1. This reveals the dominance of the elastic over the viscous behavior. Anchote starch gels can be classified as “true” gels due to their low (tan δ)_1_ values [33], ranging from 0.15–0.19 (Table 4). The (tan δ)_1_ values of PS and CS gels were significantly higher (0.36 and 0.42, respectively), denoting their lower elastic-like behavior than that of the anchote starch gels. PS and CS gels can be classified rheologically as typical weak gels [33,34].

The viscoelastic properties of anchote starch gels depended significantly (*p* < 0.05) on the cultivar. The G_1_′ and G_1_″ ranged from 168–202 Pa and 28–39 Pa, respectively, with the most consistent gels, with the highest viscoelastic moduli, being those made from D24S and REDS. The anchote starch gels, regardless of the cultivar, had G_1_′ values that were six and ten times higher than those corresponding to the PS and CS gels, respectively. This means that anchote starches are appropriate ingredients for preparing gel-like products. The dependency of the elastic and viscous moduli with frequency (*a* and *b* exponents) was also significantly lower in anchote starch gels than in PS and CS gels, denoting a more stable consistency of anchote gels versus frequency. It was reported that the rheological properties of starch gels were influenced by amylose content, branch chain length distribution of amylopectin, and starch variety [35,36]. In the case of anchote starch gels, a positive correlation between the amylose content and the G_1_′ and G_1_″ viscoelastic moduli can be concluded, in agreement with that observed by Tangsrianugul et al. [35] for rice cultivars.

### 2.7. Thermal Properties

The thermal properties and gelatinization parameters of the four anchote cultivars, along with PS and CS starches, are summarized in Table 5. The DSC thermograms obtained showed a single endothermic peak for all studied starch samples, which was related to the starch gelatinization. The peak of the dissociation of the amylose–lipid complex was not observed. The onset (T_o_), peak (T_p_), and conclusion (T_c_) temperature of the four cultivars of anchote starches ranged from 60.97 °C (WHTS) to 61.74 °C (D24S), from 63.86 °C (WHTS) to 65.07 °C (D24S), and from 67.21 °C (WHTS) to 69.33 °C (D24S), respectively. These results show significant (*p* < 0.05) variation among the cultivars of anchote starches. Abera et al. [5] reported that the gelatinization temperatures of anchote starch were 66.58 °C (T_o_), 70.18 °C (T_p_), and 73.98 °C (T_c_), which were higher than those in our results, while the gelatinization temperatures of PS and CS starches were 59.42 °C and 64.7 °C (for T_o_), 63.45 °C and 70.76 °C (for T_p_), 69.16 °C and 81.17 °C (for T_c_), respectively. The gelatinization temperatures of anchote starches showed comparable results with those for PS starches. However, the CS starch showed significantly (*p* < 0.05) higher values than the anchote and PS starches; this could be due to the crystalline structure of CS starch (A-type), since it was reported that A-type starch has a higher gelatinization temperature than B-type starch, due to a closely packed double-helices and low water content [3,22]. The gelatinization temperature range (ΔT) of studied anchote starches varied from 6.16 °C (D01S) to 7.59 °C (D24S), while the ranges of the PS (9.74 °C) and CS (16.47 °C) were significantly (*p* < 0.05) higher than those for anchote starches. The range between the onset and conclusion temperatures of the gelatinization peak represents the homogeneity degree of crystallites [12]. Therefore, a higher homogeneity in the anchote starch crystallites can be concluded, while the lowest homogeneity could be expected in CS. The gelatinization enthalpy, ΔH, reflects the energy needed to disrupt the molecular order associated with the ordered helical structure of starch [12]. The ΔH values of anchote starches varied significantly (*p* < 0.05) with the cultivar in the range of 16.87 J/g to 18.38 J/g. The highest value was obtained in WHTS, while the lowest value was found in REDS. The ΔH of anchote starches from the four cultivars was always above that of CS and similar to PS. This denotes a more stable crystalline structure in anchote starch granules, requiring higher energy to destroy it. The thermal properties of starches are altered for several reasons, including amylose content, granule size, and crystalline structure [14,22]. The variations in thermal properties observed in this study could also be due to the differences in amylose content, granule size, and crystalline structure of anchote cultivars, potato, and cassava starches [14,22].

### 2.8. Fourier Transform Infrared Spectroscopy (FTIR)

**Figure 6:**
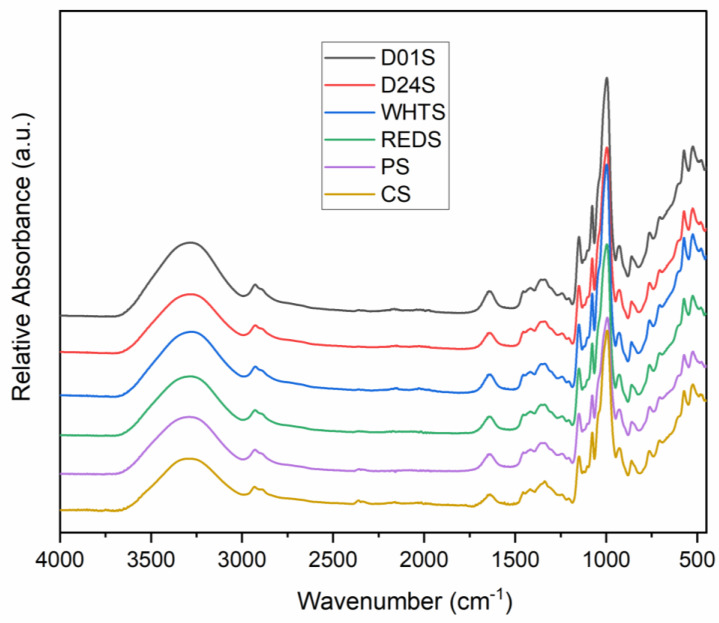
FTIR spectra of starches from four cultivars of anchote (D01S = Desta 01 starch, D24S = Desta 24 starch, WHTS = white starch, and REDS = red starch). Potato (PS) and cassava (CS) starches are included as references.

FTIR spectra of four anchote cultivars, potato, and cassava starch are presented in Figure 6. Similar spectra were observed for all studied starch samples; however, a variation in the intensities was observed. The broad band observed between 3600 and 3000 cm^−1^ indicates the O–H bond stretching of the starches. The maximum of this band was detected at different wavenumbers, depending on the anchote cultivar: 3284.49, 3281.12, 3277.61, and 3288.71 cm^−1^, for D01S, D24S, WHTS, and REDS, respectively. The weak peak observed at ~2900 cm^−1^ is related to C–H stretching. The characteristic peaks that represent the H–O–H bending vibrations of tightly bound water molecules in starch samples were observed between 1634.96 cm^−1^ (the minimum wavenumber, obtained for WHTS), and 1644.83 cm^−1^ (the maximum wavenumber, obtained for REDS).

The peaks associated with O–C–H, C–C–H, and C–O–H vibrations were observed for all studied starch samples between 1337.40 cm^−1^ (D24S) and 1338.25 cm^−1^ (REDS); the value was in accordance with a previous report [7]. A strong peak for all samples was observed at ~1000 cm^−1^, specifically for anchote cultivars (996.27 to 996.78 cm^−1^), potato (993.12 cm^−1^), and cassava (995.12 cm^−1^) starches. This peak was believed to be a characteristic of C–O bond stretching [7]. The peaks obtained between the spectral ranges 1200–1000 cm^−1^ are related to C−O, C−C, and C−O−H stretching and C−O−H bending [37]; two peaks were observed in this range in all studied starch samples. The present study reveals that the starches from four cultivars of anchote showed similar characteristic peaks having slightly variable intensities. The difference in intensity observed could be due to the amount of amylose and amylopectin present in the starch sample. Anchote starch also exhibits similar characteristic peaks compared to those of potato (PS) and cassava (CS) starches.

## 3. Conclusions

In this study, the physicochemical, morphological, thermal, and rheological properties of native starches isolated from four cultivars of anchote tubers were studied and compared to potato (PS) and cassava (CS) starches, which were considered as references. A significant impact of the cultivar on the properties of the studied anchote starches was concluded. The phosphorus content of the studied anchote starch was considerably higher than that of the reference starches, favoring the functional characteristics of the starch due to its enhanced paste viscosity, clarity, and lightness, making it a possible alternative to potato starch. The result shows the amylose contents of starches from anchote cultivars were within the range reported for most root and tuber starches, and these were categorized in the range of normal/regular starch. Similar morphologies were observed among the studied cultivars of anchote starch granules; however, they had different particle sizes. The four cultivars of anchote and PS starches had a B-type crystalline structure, while CS starch had an A-type crystalline structure. The results of pasting properties showed that anchote starch had a higher peak viscosity than CS starch, but a lower peak viscosity than PS starch. The starches of anchote cultivars showed higher stability to heating and shearing, exhibiting a higher trough and final viscosity than PS and CS starches, which are important characteristics for products that require a higher stability of the gel after cooling, making it suitable for applications that require higher viscosities. Anchote starch gels showed rheological characteristics of true gels, with significantly higher elastic behavior (lower tan δ) and stronger consistency (much higher viscoelastic moduli) than those found in CS and PS gels. Therefore, the results revealed that anchote starches are suitable ingredients for preparing gel-like products. Therefore, the present study reveals the promising potential utilizations of anchote starch in as a functional ingredient in food processing, product development, novel food product formulations, and other industrial applications, particularly in the formulation of gel-like products.

## 4. Materials and Methods

### 4.1. Materials

Two anchote tubers Desta 01 (D01) and Desta 24 (D24) were collected from Debre Zeit Agricultural Research Center (DZARC), Bishoftu, Ethiopia. The Desta 01 is the only released variety of anchote, while Desta 24 is a promising accession to be released as a second variety. The other two types of local anchote cultivar tubers, (white, WHT, and red, RED, were collected from local growers around Metu, in the western part of Ethiopia. The potato (PS) and cassava (CS) starch used in this study as references were supplied by Ferrer Alimentación S.A. (Barcelona, Spain), and Cargill S.L. (Brenntag, Seville, España), respectively.

### 4.2. Isolation of Starch

The starch was isolated from anchote tubers according to the methods of Falade and Okafor [38], with slight modifications. The collected anchote tubers were sorted and cleaned by washing with water to remove any dust and soils from the surface and were then peeled by hand. The peeled anchote tubers were sliced into small cubes and mixed with distilled water at a ratio of 1:10 (*v*/*v*) for 4 min using a laboratory-grade blender (Silver crest multifunctional blender, Weifang, China). The slurry was then filtered using a fine muslin cloth. The filtrate was allowed to stand for 14 h for sedimentation, the supernatant was drained, and the residue was washed repeatedly with distilled water until the color became white. The obtained clear sediment was dried at 45 °C for 24 h in a drying oven. The dried starch was ground, packed in a zip-locked polythene bag, and kept in a cool, dry place for further analysis. Anchote starch samples were named: D01S = Desta 01 starch, D24S = Desta 24 starch, WHTS = white starch, and REDS = red starch.

### 4.3. Starch Particle Size, Crystallinity, and Morphology

The particle size of anchote starches was analyzed by a laser diffraction particle size analyzer (Mastersizer 3000, Malvern Instruments Ltd., Malvern, UK). The results were obtained as the volume distribution and mean diameter of starch granules, which were expressed by the median diameter (D_50_) and the dispersion (D_90_ − D_10_)/D_50_, as described in [39], and the surface-weighted mean diameter [D(3,2)] and the volume-weighted mean diameter [D(4,3)] as described by [14]. The measurements were carried out in triplicate.

The X-ray diffraction patterns (XRD) of the starch samples were studied using a Bruker-D8-Discover-A25 diffractometer (Bruker AXS, Rheinfelden, Germany) following the method previously described by Vela et al. [40]. The XRD was equipped with a copper tube (Cu-Kα radiation, wavelength of 0.154 nm) which operates at 40 mA and 40 kV. The starch samples were equilibrated at 15% humidity before the analysis. The spectra of the equilibrated starch samples were obtained in the range of 5°–40° of the 2θ diffraction angle and at a speed of 1.2°/min. The relative crystallinity (%) was obtained as the ratio between the diffraction peak to the total diffraction area using DifracEVA software with PDF2-2004 and Crystallography Open Database.

The micromorphology of the starch samples was examined using a Quanta 200FEG scanning electron microscope (FEI, Corvallis, OR, USA), equipped with an X-ray detector, which allowed for the study of the starch samples without metallization [40]. The samples were directly mounted on stubs and observed with an accelerating voltage of 5 keV in low vacuum mode by a secondary electron detector. The micrographs of anchote, potato, and cassava starches were captured at various magnifications (1000×, 4000×, and 8000×).

### 4.4. Proximate and Mineral Composition

The moisture, ash, and fat content of anchote, potato, and cassava starches were determined using the standard methods of the Association of Official Analytical Chemists (AOAC) [41]. The nitrogen content was determined by an elemental CNS-analyzer (Elemental Analyzer LECO CNS928). The nitrogen content found in the starch samples was converted to protein content by using a 6.25 conversion factor [14]. The phosphorus and calcium contents were determined following the method of Ronda et al. [42], using a radial simultaneous inductively coupled plasma optical emission spectrometry (ICP-OES) Varian 725-ES spectrophotometer (Agilent Technologies, Santa Clara, CA, USA).

### 4.5. Amylose and Total Starch Determination

The amylose and total starch content of isolated anchote starches, as well as potato and cassava starches, were determined using the Megazyme amylose/amylopectin assay kit (Megazyme, Bray Business Park, Bray, Ireland), according to the manufacturer’s instructions.

### 4.6. Pasting Properties

Pasting properties were determined using the Rapid Visco Analyser (RVA 4500, Perten Instruments, PerkinElmer, Sydney, Australia) following the RVA method for potato starch (RVA Method 07.04). Briefly, two grams of starch (at 14% moisture basis) were weighed in the RVA canister and mixed with 25 mL of distilled water. The prepared starch-water slurry was subjected to RVA analysis, where it was analyzed under programmed heating and cooling conditions. The starch-water slurry was mixed at 960 rpm for the first 10 s, and then at 160 rpm throughout the analysis. The programmed temperature-time variation was: holding for 1 min at 50 °C, heating gradually to 95 °C at 12 °C/min, holding for 2.5 min at 95 °C, cooling down at 12 °C/min up to 50 °C, and finally holding at 50 °C for 2 min. The pasting property parameters recorded were peak viscosity (PV), trough viscosity (TV), breakdown viscosity (BV), setback viscosity (SV), final viscosity (FV), pasting temperature (PT), and peak time (P time). All samples were analyzed in triplicate.

### 4.7. Rheological Properties

The fresh gels of starch samples obtained from the pasting property analysis described in Section 4.6 above were used for the determination of the gel rheological properties. The rheological tests were performed with a Kinexus Pro+ rheometer (Malvern Instruments Ltd., Malvern, UK) using a serrated parallel plate geometry of 40 mm diameter and a measuring gap of 1 mm. The anchote gels were placed on the bottom of the plate and left to rest for 5 min to allow sample relaxation. Strain sweeps were conducted from 0.10 to 1000% strain at a constant frequency of 1 Hz. Frequency sweeps were carried out from 10 to 1 Hz by applying a constant strain of 0.5%, which was within the linear viscoelastic region (LVR). The tests were conducted at a constant temperature (25°C). The rheological data were analyzed using rSpace rheometry software for Kinexus V1.72 (Malvern Instruments Ltd., UK). Frequency sweep data were fitted to the power-law model, as previously described by Ronda et al. [43]. The recorded viscoelastic parameters, G_1_′, G_1_″, and (tan δ)_1_, represent the elastic and viscous moduli and the loss tangent, respectively, at a frequency of 1 Hz. Then, *a, b*, and *c,* are the exponents of the corresponding potential equations (power-law model) and quantify the dependence of the dynamic moduli on the oscillation frequency. All tests were carried out in triplicate.

### 4.8. Thermal Properties

The thermal properties of the starch samples were measured by differential scanning calorimetry (DSC3, STAR^e^ System, Mettler Toledo, Switzerland) according to the method utilized in Vela et al. [40]. Anchote starch samples (~6 mg) were weighed into 40 μL aluminum pans, and distilled water was added to reach the ratio of 30:70 (w:w, starch: water). The pans were sealed and kept at room temperature for 30 min before the analysis. The sealed pans were scanned from 0 to 110 °C at a rate of 5 °C/min, using an empty sealed pan as a reference. The measured gelatinization properties were: onset (T_o_), peak (T_p_), and conclusion (T_c_) temperatures (°C), and the enthalpy of gelatinization (ΔH) (J/g dry starch). All samples were measured in duplicate.

### 4.9. Fourier Transform Infrared Spectroscopy (FTIR)

The functional group analysis of anchote starch and control starches (potato and cassava) was performed using an FTIR Nicolet iS50 spectrophotometer (Thermo Fisher Scientific, Waltham, MA, USA) coupled with an attenuated total reflectance (ATR) device equipped with a diamond crystal. The starch samples were equilibrated at a humidity of 15% following the method previously used by Vela et al. [40]. The powdered starch sample was placed on a diamond crystal and pressed until the desired level was reached. The spectra were scanned from 400–4000 cm^−1^ with a resolution of 4 cm^−1^, and a total of 64 scans were measured and averaged. Each sample was scanned in triplicate, and the obtained spectra were baseline corrected and normalized using OMNIC software (Thermo Fisher Scientific, Waltham, MA, USA).

### 4.10. Statistical Analysis

The results were examined using a one-way analysis of variance (ANOVA), and Tukey’s honest significant difference (HSD) test was used to assess significant differences at *p* < 0.05 using Statgraphics Centurion v.18 (Bitstream, Cambridge, MN, USA.). All the results were reported as means and corresponding standard deviations.

## Figures and Tables

**Table 1:** Particle size distribution of four cultivars of anchote, potato, and cassava starches.

Sample	D_50_ (μm)	D [3,2] (μm)	D [4,3] (μm)	(D_90_ − D_10_)/D_50_
D01S	11.6 ± 0.1 a	12.0 ± 0.1 a	63.9 ± 3.3 c	20.6 ± 1.2 c
D24S	14.4 ± 0.1 bc	13.5 ± 0.1 bc	66.4 ± 2.7 c	15.5 ± 0.4 b
WHTS	14.5 ± 0.7 c	14.7 ± 0.6 c	106.9 ± 7.8 e	22.5 ± 0.3 d
REDS	13.3 ± 0.4 b	13.6 ± 0.5 bc	81.7 ± 4.2 d	20.8 ± 0.2 c
PS	36.2 ± 0.6 d	31.9 ± 0.7 d	39.5 ± 0.6 b	1.3 ± 0.1 a
CS	13.5 ± 0.1 bc	12.5 ± 0.1 ab	14.3 ± 0.1 a	1.0 ± 0.1 a

D_50_: median diameter, (D_90_ − D_10_)/D_50_ size dispersion; D [3,2]: surface-weighted mean diameter; D [4,3]: volume-weighted mean diameter. D01S = Desta 01 starch, D24S = Desta 24 starch, WHTS = white starch, REDS = red starch, PS = potato starch, and CS = cassava starch. Data are the mean ± standard deviation (n = 3). Values in the same column followed by the same letter are not significantly different (*p* < 0.05).

**Table 2:** Proximate composition; total starch; and amylose, phosphorus, and calcium content of anchote cultivars, potato, and cassava starches. All results, except moisture, are referred to as the weight of dry matter.

Sample	Moisture (%)	Ash (%)	Fat (%)	Protein (%)	Total Starch (%)	Amylose (%)	Phosphorus (mg/100 g)	Calcium (mg/100 g)
D01S	20.24 ± 0.01 f	0.38 ± 0.01 c	0.09 ± 0.01 b	0.65 ± 0.01 c	80.8 ± 1.0 cd	15.8 ± 0.9 a	82. 8 ± 0.6 c	45.8 ± 0.4 e
D24S	17.79 ± 0.02 c	0.39 ± 0.01 d	0.14 ± 0.01 d	0.64 ± 0.02 bc	74.9 ± 0.2 b	22.3 ± 0.6 c	83.2 ± 0.1 c	55.7 ± 0.5 f
WHTS	19.41 ± 0.03 e	0.47 ± 0.01 e	0.10 ± 0.01 bc	0.51 ± 0.03 b	77.6 ± 1.8 bc	17.2 ± 0.1 ab	93.3 ± 2.4 d	30.4 ± 0.5 c
REDS	18.52 ± 0.01 d	0.49 ± 0.01 f	0.11 ± 0.01 c	0.54 ± 0.07 bc	66.8 ± 1.9 a	22.3 ± 0.2 c	92.2 ± 1.3 d	36.8 ± 0.4 d
PS	14.52 ± 0.01 b	0.32 ± 0.01 b	0.09 ± 0.01 b	0.32 ± 0.01 a	84.1 ± 1.6 d	18.8 ± 0.5 b	60.3 ± 0.3 b	7.4 ± 0.1 a
CS	12.78 ± 0.01 a	0.18 ± 0.01 a	0.02 ± 0.01 a	0.36 ± 0.02 a	96.2 ± 1.1 e	19.0 ± 0.4 b	5.8 ± 0.1 a	26.3 ± 0.1 b

D01S = Desta 01 starch, D24S = Desta 24 starch, WHTS = white starch, REDS = red starch, PS = potato starch, and CS = cassava starch. Data are the mean ± standard deviation (n = 2). Values in the same column followed by the same letter are not significantly different (*p* < 0.05).

**Table 3:** Pasting properties of anchote cultivars, potato, and cassava starches.

Sample	PV (mPa·s)	TV (mPa·s)	BV (mPa·s)	FV (mPa·s)	SV (mPa·s)	Ptime (min)	PT (°C)
D01S	2726 ± 20 d	2239 ± 22 c	487 ± 28 bc	3466 ± 15 d	1228 ± 23 b	4.89 ± 0.10 c	70.33 ± 0.08 b
D24S	2293 ± 45 b	2046 ± 47 b	248 ± 54 a	3686 ± 14 e	1640 ± 43 d	5.29 ± 0.04 e	72.65 ± 0.05 c
WHTS	2649 ± 53 d	2280 ± 38 c	369 ± 27 ab	3495 ± 21 d	1215 ± 35 b	5.33 ± 0.01 e	69.38 ± 0.06 a
REDS	2448 ± 28 c	2058 ± 44 b	389 ± 35 b	3409 ± 20 c	1351 ± 32 c	5.15 ± 0.04 d	69.5 ± 0.09 a
PS	5728 ± 26 e	1942 ± 69 b	3786 ± 87 d	2322 ± 21 b	380 ± 49 a	3.27 ± 0.01 a	68.83 ± 0.45 a
CS	1367 ± 38 a	796 ± 14 a	571 ± 24 c	1222 ± 15 a	426 ± 10 a	4.47 ± 0.01 b	75.65 ± 0.44 d

PV = peak viscosity, TV = trough viscosity, BV = breakdown viscosity, SV = setback viscosity, FV = final viscosity, PT = pasting temperature, and Ptime = peak time. D01S = Desta 01 starch, D24S = Desta 24 starch, WHTS = white starch, REDS = red starch, PS = potato starch, and CS = cassava starch. Data are the mean ± standard deviation (n = 3). Values in the same column followed by the same letter are not significantly different (*p* < 0.05).

**Table 4:** Rheological properties of anchote, potato, and cassava starches.

Sample	G_1_′ (Pa)	*a*	G_1_′′ (Pa)	*b*	(tan δ)_1_	*c*	Cross Point (Pa)	τ_max_ (Pa)
D01S	169 ± 3 c	0.11 ± 0.01 a	28.1 ± 0.6 c	0.29 ± 0.01 a	0.17 ± 0.01 b	0.18 ± 0.01 b	285 ± 1 b	181 ± 3 d
D24S	202 ± 8 d	0.12 ± 0.01 a	39.1 ± 1.6 d	0.28 ± 0.01 a	0.19 ± 0.01 c	0.16 ± 0.01 b	281 ± 6 b	171 ± 7 c
WHTS	175 ± 2 c	0.11 ± 0.01 a	28.7 ± 0.2 c	0.28 ± 0.01 a	0.16 ± 0.01 b	0.17 ± 0.01 b	243 ± 7 a	131 ± 2 a
REDS	198 ± 4 d	0.10 ± 0.01 a	30.1 ± 0.9 c	0.28 ± 0.01 a	0.15 ± 0.01 a	0.18 ± 0.01 b	242 ± 1 a	151 ± 2 b
PS	34 ± 1 b	0.22 ± 0.01 b	12.3 ± 0.6 b	0.32 ± 0.01 b	0.36 ± 0.01 d	0.11 ± 0.01 a	nd	nd
CS	19 ± 1 a	0.24 ± 0.02 c	8.0 ± 0.1 a	0.35 ± 0.01 c	0.42 ± 0.01 e	0.11 ± 0.02 a	nd	nd

D01S = Desta 01 starch, D24S = Desta 24 starch, WHTS = white starch, REDS = red starch, PS = potato starch, and CS = cassava starch. Data are the mean ± standard deviation (n = 3). Values in the same column followed by the same letter are not significantly different (*p* < 0.05). G_1_′, G_1_′′, and (tan δ)_1_ were obtained from fitting the frequency sweep data to the power model and represent the elastic modulus, viscous modulus, and loss tangent at a frequency of 1 Hz, respectively. The a, b, and c exponents quantify the dependence degree of dynamic moduli and the loss tangent with the oscillation frequency. τ_max_ = the maximum stress that the samples can tolerate in the linear viscoelastic region (LVR). The cross point represents the stress where the G’ = G’’. nd: not detectable in the studied strain range.

**Table 5:** Thermal properties of anchote, potato, and cassava starches.

Sample	T_O_ (°C)	T_p_ (°C)	T_C_ (°C)	ΔT (°C)	ΔH (J/g)
D01S	61.13 ± 0.15 b	64.11 ± 0.12 b	67.28 ± 0.23 a	6.16 ± 0.08 a	17.16 ± 0.46 b
D24S	61.74 ± 0.09 c	65.07 ± 0.07 c	69.33 ± 0.16 c	7.59 ± 0.07 b	18.01 ± 0.47 bc
WHTS	60.97 ± 0.09 b	63.86 ± 0.22 ab	67.21 ± 0.23 a	6.25 ± 0.13 a	18.38 ± 0.47 bc
REDS	61.34 ± 0.08 bc	64.31 ± 0.15 b	67.97 ± 0.07 b	6.64 ± 0.01 a	16.87 ± 0.99 ab
PS	59.42 ± 0.04 a	63.45 ± 0.11 a	69.16 ± 0.09 c	9.74 ± 0.05 c	18.75 ± 0.78 c
CS	64.7 ± 0.28 d	70.76 ± 0.07 d	81.17 ± 0.04 d	16.47 ± 0.25 d	15.44 ± 0.24 a

ΔH = enthalpy of gelatinization; T_O_ = onset transition temperature; T_p_ = peak transition temperature; T_c_ = conclusion transition temperature; ΔT = gelatinization temperature range (T_c_ − T_o_). D01S = Desta 01 starch, D24S = Desta 24 starch, WHTS = white starch, REDS = red starch, PS = potato starch, and CS = cassava starch. Data are the mean ± standard deviation (n = 2). Values in the same column followed by the same letter are not significantly different (*p* < 0.05).
